# Unexpected non-reactive nontreponemal serology in secondary syphilis: The prozone phenomenon and clinician–laboratory communication

**DOI:** 10.4102/sajid.v41i1.800

**Published:** 2026-04-24

**Authors:** Mahomed A. Aboo, Matilda Mphahlele, Melanie Louw, Nicholas Aikman

**Affiliations:** 1 Department of Internal Medicine, Tambo Memorial Hospital, Johannesburg, South Africa; 2 Department of Dermatology, Tambo Memorial Hospital, Johannesburg, South Africa; 3 Department of Anatomical Pathology, Charlotte Maxeke Johannesburg Academic Hospital, National Health Laboratory Service, Johannesburg, South Africa; 4 Department of Anatomical Pathology, School of Pathology, Faculty of Health Sciences, University of the Witwatersrand, Johannesburg, South Africa

**Keywords:** secondary syphilis, prozone phenomenon, syphilis serology, serological discordance, *Treponema pallidum*

## Abstract

We describe a case of a 22-year-old woman living with human immunodeficiency virus (HIV) who presented with classical features of secondary syphilis. Initial Rapid Plasma Reagin (RPR) testing was unexpectedly non-reactive; however, repeat RPR testing with serum dilution yielded a reactive result, illustrating the prozone phenomenon.

**Contribution:**

The prozone phenomenon is uncommon. We aim to encourage clinician–laboratory communication when there is a high pretest probability of syphilis, with serological discordance, to facilitate prompt diagnosis and treatment.

## Introduction

Syphilis is an infectious disease caused by the spirochaete *Treponema pallidum* subspecies *pallidum* (*T. pallidum*). It is transmitted predominantly through sexual activity or vertically from mother to child, resulting in congenital syphilis. Since the introduction of syndromic management of sexually transmitted infections in South Africa, the prevalence of syphilis has reduced.^1^ However, more recent studies have shown a resurgence of syphilis.^2,3^

The laboratory diagnosis of syphilis is primarily serological and relies on a combination of treponemal and nontreponemal tests. It is important to note that nontreponemal tests can yield unexpectedly non-reactive results, occasionally as a consequence of the prozone phenomenon.^4,5,6,7^ Detection of the prozone phenomenon is clinically significant when there is a high pretest probability of syphilis, but unexpectedly non-reactive nontreponemal serology. In such instances, clinician–laboratory communication should prompt further diagnostic evaluation, including serum dilution. We describe a case of a 22-year-old woman living with human immunodeficiency virus (HIV), wherein the prozone phenomenon was evident.

## Case

A 22-year-old woman living with HIV presented with a 2-month history of a body rash. She admitted to interrupting her antiretroviral therapy 2 months before presenting to the hospital. Upon further enquiry, she reported a history of a painless genital ulcer approximately 3 weeks before the onset of the body rash. The ulcer had spontaneously resolved. The patient was haemodynamically stable and apyrexial. On general examination, she had cervical lymphadenopathy. The rash consisted of polymorphous skin lesions, including hyperpigmented macules, violaceous plaques with surface scale and violaceous macules and plaques with collarette scale.

**FIGURE 1:**
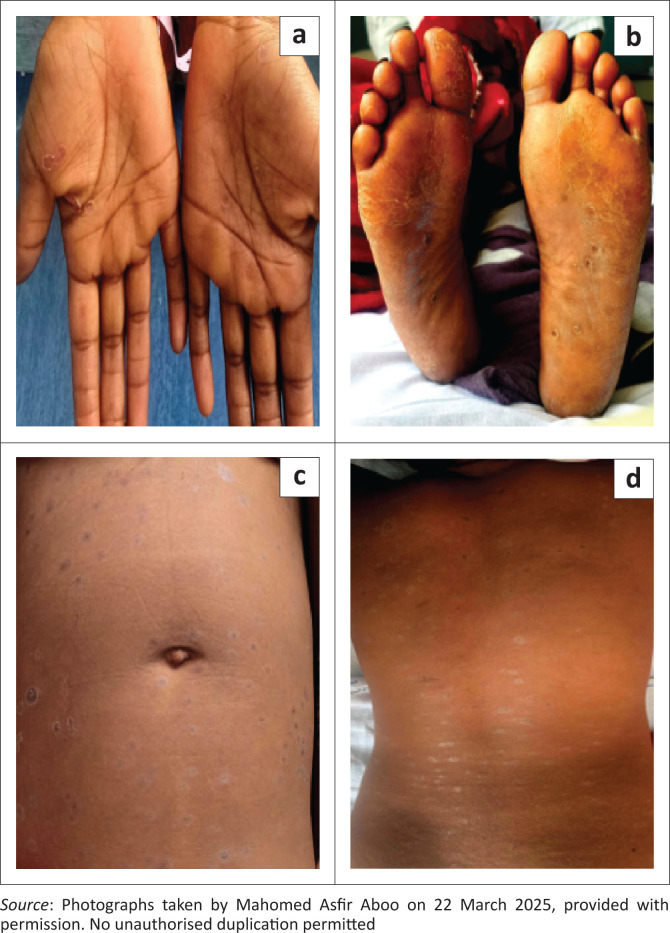
The rash of secondary syphilis on the palms (a), soles (b) and torso (c) & (d) of the patient.

The lesions involved the patient’s trunk, limbs, palms and soles. These are depicted in Figure 1. The patient had a cluster of differentiation 4 (CD4) count of 500 cells/µL and an HIV viral load of 248 copies/mL. Clinically, secondary syphilis was suspected, and syphilis serological tests were requested. The tests were performed at a National Health Laboratory Service facility using the reverse sequence algorithm. The *Treponema pallidum* antibody (TPAB) test, an automated electrochemiluminescent immunoassay (Roche Elecsys Syphilis assay), was reactive, but the Rapid Plasma Reagin (RPR) test was non-reactive. Despite the non-reactive RPR test, the treating clinicians had a high index of suspicion for secondary syphilis and so considered the possibility of an unexpectedly non-reactive RPR result as a consequence of the prozone phenomenon.

**FIGURE 2:**
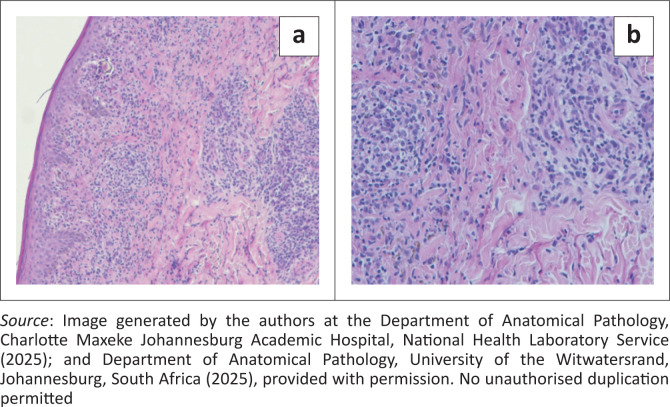
**(a and b):** Haematoxylin and Eosin staining (100X and 400X, respectively) Sections show epidermis and dermis in which there is a dense chronic inflammatory cell infiltrate. The infiltrate comprises lymphocytes, histiocytes and plasma cells. A prominent focus of perivascular chronic inflammatory cell infiltration is evident in the reticular dermis and papillary dermis, with exocytosis of lymphocytes into the epidermis.

**FIGURE 3:**
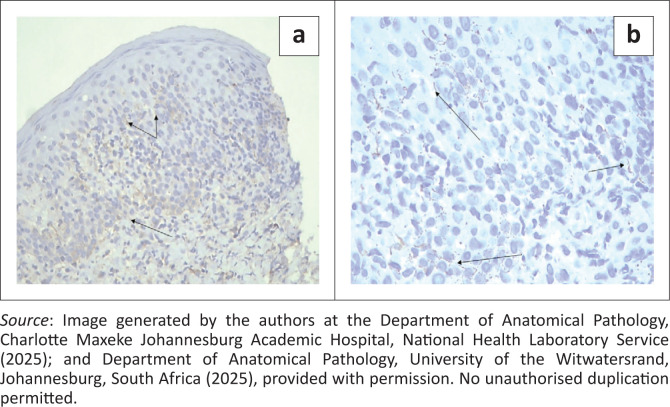
**(a and b):** *T. pallidum* Immunoperoxidase Staining (200X and 400X, respectively) Positive staining of *T. pallidum* spirochaetes in the epidermis and dermis.

Consequently, a skin biopsy was done, and a second blood sample was collected within 24 h of the initial specimen and submitted for repeat RPR testing with serum dilution following clinician–laboratory communication. The skin biopsy confirmed secondary syphilis as demonstrated in Figure 2 and Figure 3, and the repeat RPR test with serum dilution was reactive with a titre of 512. The patient was treated with a single dose of Benzathine Penicillin G 2.4 million units intramuscularly according to the Southern African HIV Clinicians Society Guideline,^1^ and the rash subsequently resolved.

## Discussion

Syphilis can broadly be classified into early and late syphilis. Early syphilis includes primary, secondary and early latent syphilis. Late syphilis includes tertiary syphilis and late latent syphilis.^8^ Primary syphilis is characterised by an ulcerated lesion known as a chancre, which is often painless. Chancres are most commonly found in the genital area, but can occur at any site where exposure and inoculation have occurred, such as the fingers, nipples and mucosal surfaces of the mouth.^9^ Secondary syphilis most commonly presents with a mucocutaneous eruption consisting of multiple red-brown macules and papules involving the palms, soles, trunk and extremities, which is often associated with lymphadenopathy.^9^ Tertiary syphilis presents with cardiovascular involvement or gummatous disease.^6^ Latent syphilis refers to the presence of serological evidence of active infection in an asymptomatic individual.^8^ Latent syphilis can be early, if diagnosed within 12 months of infection with *T. pallidum*, or late, if diagnosed more than 12 months after infection with *T. pallidum*.^5,6^ Neurosyphilis can occur at any stage of the disease.^5,6^

Due to the variability in the presentation of syphilis, laboratory testing is necessary. Laboratory tests can either be direct detection methods or serology. Direct detection methods include nucleic acid amplification tests, dark-field microscopy and histology, but are costly, require specialised equipment and technical expertise.^4,5^ Serology is widely available and therefore is the primary diagnostic modality for syphilis. Serology can be classified into treponemal and nontreponemal tests.^4,5^

Treponemal tests are qualitative tests that detect the presence of immunoglobulin G and immunoglobulin M (IgG and IgM) antibodies against *T. pallidum*. They cannot be used to monitor response to treatment, nor can they be used to detect re-infection. Once a treponemal test is reactive, it remains reactive indefinitely despite treatment.^4^

Examples of treponemal tests commonly available in South Africa are the enzyme immunoassay, chemiluminescent immunoassay IgG/IgM, fluorescent treponemal antibody-absorption test, *T. pallidum* Haemagglutination Assay (TPHA) and the *T. pallidum* Particle Agglutination Assay (TPPA).^1^

Nontreponemal tests are quantitative assays that report antibody titres. They are used to assess disease activity, monitor treatment response and detect re-infection.^4^ Examples of common nontreponemal tests are the RPR test and the Venereal Disease Research Laboratory (VDRL) test.^1^ Nontreponemal tests are flocculation assays that detect a lattice formed by antigen-antibody complexes.^6^ These assays can yield unexpectedly non-reactive results under certain circumstances. One such limitation is the prozone phenomenon, in which excess antibodies interfere with optimal lattice formation, resulting in apparent non-reactivity.^4,5,6,7^

Reported associations of the prozone phenomenon include patients with secondary syphilis and certain clinical populations such as individuals living with HIV.^10,11^ Nevertheless, the incidence is low, estimated at 0.2% – 2%.^5,12,13^

There are unfortunately no studies that have measured the incidence of the prozone phenomenon in South African cohorts. While HIV infection has been hypothesised to increase the risk of the prozone phenomenon through immune dysregulation and excess antibody production,^11,14^ this theory originates from literature early in the HIV era and has not been consistently substantiated. Moreover, the prozone phenomenon is more likely to be detected in secondary syphilis as patients often present with florid clinical features, and a non-reactive nontreponemal test will prompt further investigation. Consequently, prozone phenomena are more likely to go undetected in other stages of the disease. Reported associations may therefore be influenced by detection and publication bias.

**FIGURE 4:**
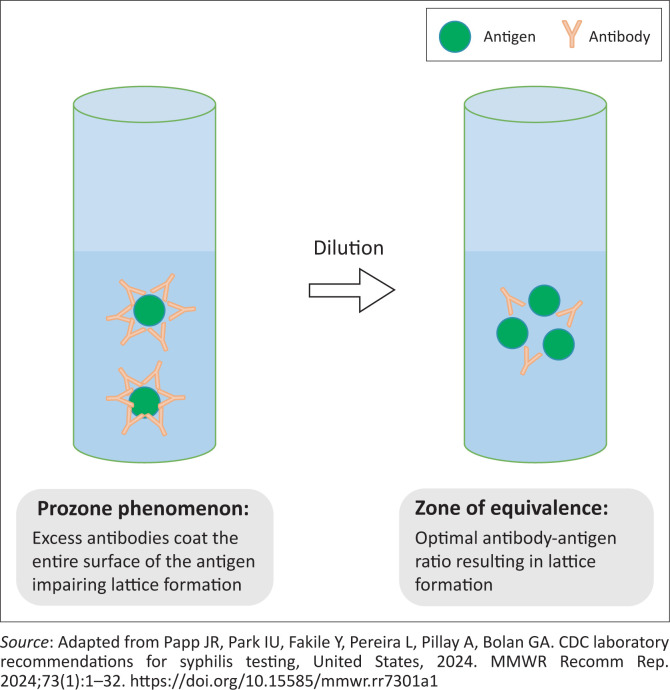
Interactions between antibodies and antigens in nontreponemal tests.^6^

The clinical significance of the prozone phenomenon lies in the possibility of an unexpectedly non-reactive nontreponemal test in a patient with a high pretest probability of syphilis. In such instances, the diagnosis of syphilis should not be excluded by virtue of a single non-reactive nontreponemal test. Instead, the nontreponemal test may need to be repeated with serum dilution following clinician–laboratory communication. Serum dilution optimises the ratio of antibodies to antigens required for lattice formation, thus restoring test reactivity.^4,5,6,7^ This optimal ratio is known as the zone of equivalence.^6^ The prozone phenomenon and the zone of equivalence are demonstrated in Figure 4. However, routine dilution of all nontreponemal tests is not practical since the prozone phenomenon is rare, and since no set range of dilutions has been determined to detect it.^6,7^ Targeted dilution based on a high clinical suspicion is therefore more reasonable.

Furthermore, using the reverse sequence algorithm (in which a treponemal test is performed first, if reactive, a nontreponemal test is then performed) as opposed to the traditional sequence algorithm (in which a nontreponemal test is performed first and only if reactive, a treponemal test is then performed) has the advantage of detecting serological discordance.^15^

Interpretation of serological discordance requires a structured diagnostic framework comprising of clinical factors, pre-analytical factors, analytical factors and post-analytical factors. Clinical factors include the stage of the disease (particularly early infection with seroconversion if there is a temporal gap between specimens) and previous syphilis infection.^4,6,15^ Preanalytical factors include specimen mislabelling and misidentification.^16^

Analytical factors include the prozone phenomenon and false-positive treponemal results.^15^ Post-analytical factors include laboratory transcription and reporting errors.^16^ Therefore, careful clinical assessment, review of specimen handling and clinician–laboratory communication are essential when interpreting discordant results.

## Conclusion

The prozone phenomenon can present a diagnostic dilemma. Undiagnosed and untreated syphilis can result in ongoing transmission of syphilis, an increased risk of HIV transmission or acquisition, as well as progression of syphilis to advanced disease, which is associated with significant morbidity and mortality.^8,17^ As there is a resurgence of syphilis in South Africa, knowledge of the prozone phenomenon is crucial to facilitate accurate diagnosis and timely treatment. We highlight the importance of maintaining a high index of suspicion, using the reverse sequence algorithm and repeating nontreponemal tests with serum dilution, guided by clinician–laboratory communication, to increase detection of the prozone phenomenon.
